# Bioremediation and Detoxification of the Textile Wastewater with Membrane Bioreactor Using the White-rot Fungus and Reuse of Wastewater

**DOI:** 10.15171/ijb.1216

**Published:** 2016-09

**Authors:** Kaizar Hossain, Shlrene Quaik, Norli Ismail, Mohd Rafatullah, Maruthi Avasan, Rameeja Shaik

**Affiliations:** ^1^Department of School of Industrial Technology, University Sains Malaysia, Pulau Pinang, Malaysia; ^2^Department of Environmental Studies, GITAM University, Vishakhapatnam, AP India; ^3^Department of Environmental Sciences, Andhra University, Vishakhapatnam, AP India

**Keywords:** Bioremediation, Membrane bioreactor, Textile wastewater, White-rot fungi

## Abstract

**Background:**

Application of membrane technology to wastewater treatment has expanded over the last decades due to increasingly stringent legislation, greater opportunities for water reuse/recycling processes and continuing advancement in membrane technology.

**Objectives:**

In the present study, a bench-scale submerged microfiltration membrane bioreactor (MBR) was used to assess the treatment of textile wastewater.

**Materials and Methods:**

The decolorization capacity of white-rot fungus coriolus versicolor was confirmed through agar plate and liquid batch studies. The temperature and pH of the reactor were controlled at 29±1°C and 4.5±2, respectively. The bioreactor was operated with an average flux of 0.05 m.d^-1^ (HRT=15hrs) for a month.

**Results:**

Extensive growth of fungi and their attachment to the membrane led to its fouling and associated increase of the transmembrane pressure requiring a periodic withdrawal of sludge and membrane cleaning. However, stable decoloration activity (approx. 98%), BOD (40-50%), COD (50-67%) and total organic carbon (TOC) removal (>95%) was achieved using the entire system (fungi + membrane), while the contribution of the fungi culture alone for TOC removal, as indicated by the quality of the reactor supernatant, was 35-50% and 70%, respectively.

**Conclusions:**

The treated wastewater quality satisfied the requirement of water quality for dyeing and finishing process excluding light coloration. Therefore, textile wastewater reclamation and reuse is a promising alternative, which can both conserve or supplement the available water resource and reduce or eliminate the environmental pollution.

## 1. Background

Industrial processes are causing the production of large amount of toxic and stable pollutants, which are all collected into the water outcoming from the plant. The disposal of these contaminated effluents into receiving waters can cause environmental damages, directly influencing the aquatic ecosystem and even human being. The textile industry is one of the largest water consumers and is rated as the most polluting among all industrial sectors considering both volume and composition of the effluent globally (1, 2). It is a complex and highly variable mixture of many polluting substances ranging from inorganic compounds and elements to polymers and organic products (3-5). It induces persistent color coupled with organic load leading to disruption of the total ecological/symbiotic balance of the receiving water stream. Dyes with striking visibility in recipients may significantly affect photosynthetic activity in aquatic environment due to the reduced light penetration and may also be toxic to some aquatic lives due to metals, chlorides, etc., associated with dyes or the dyeing process. It is difficult to remove dyes from effluents since dyes are stable to light, heat and oxidizing agents and are non‏ biodegradable (6, 7).

Anaerobic and aerobic microorganisms can both be useful for the treatment of the textile wastewater. Enzymes with azoreductase activity have been found in many types of aerobic and anaerobic microorganisms including bacteria, fungi, and algae. The most interesting oxidases have been identified in microorganisms that live under aerobic conditions. Anaerobic processes using consortia of microorganisms (anaerobic sludge or biofilm) as the active agents have often been used for textile wastewater treatment (8, 9). These processes are effective in reducing the organic content of the effluent and also in removing the color by reducing the azo bonds (10). Their advantage compared to aerobic processes is that they can handle higher organic loads, they do not need aeration, and they generate less sludge. On the other hand**,** the aerobic microorganisms have oxidative enzymes that can break down the aromatic amines released during anaerobic color removal.

In the past 15-20 years, white-rot fungi have been applied to different biotechnological fields for their capability to degrade many aromatic compounds. The wood rotting ‘white-rot’ fungi are able to degrade aerobically a wide variety of recalcitrant organic pollutants, including various types of dyes through extracellular secretion of non-specific oxidative enzymes as a secondary metabolic activity in C or N-limited medium (1, 11-13). The application of white-rot fungi in large-scale for wastewater treatment has been impeded by the lack of bioreactor systems that can sustain steady production of high levels of ligninolytic enzymes for a long period together with a controlled growth of fungi (14-16). The extensively used systems were moved towards tank reactor and air-lift and bubble column, fixed bed bioreactor, rotating disk reactor and the silicone-membrane reactor (17). Specifically, few reports are there on dye decolorization in continuous bioreactors such as Asgher *et al*. (2006) that have reported 80% decolorization of a disperse dye (Red-553) in a continuous (10-20 days) fixed-film bioreactor. Zhang *et al*. (1999), also investigated the continuous decolorization of an azo dye, Orange II, in a packed-bed reactor, achieving a high decolorization efficiency (97%). However, a number of operational problems such as the formation of mycelia aggregates, electrode fouling, and clogging emerged after a short time and made the periodical removal of the fungal biomass from the reactors necessary. Mielgo *et al*. (2001) have proposed a pulsed flow bioreactor packed with immobilized fungi, which treated with a dye loads of 0.2 g dye.(m^3^)^-1^ a day at over 90% efficiency for several months. *In vitro* dye decolorization by manganese peroxidase in an enzymatic membrane reactor in continuous operation has been studied by Lopez *et al*. (2002). The system allowed a very fast decoloration with over 90% efficiency under high dye loading rate of 2.4 g dye.(m^3^)^-1^.d. Fujita *et al*. (2000) could achieve to 70% decolorization of heat-treatment liquor (HTL) of the waste sludge which is the byproduct of heat treatment of sewage sludge by a bioreactor using polyurethane foam-immobilized white-rot fungus equipped with a side stream ultrafiltration membrane. The overall feasibility of such a system, however, would depend on the alleviation of the membranefouling problem. Selection and proper arrangement of an appropriate type of membrane, less vulnerable to the fungi attachment and also easier to clean (for example, a flat sheet type membrane), in a properly designed reactor ensuring efficient mass transfer with an adequate aeration system is required (23, 24).

The quality of the treated effluent output from the membrane bioreactor is more stable than that achieved by employing other techniques, enabling optimal functioning of the secondary treatment system. According to the authors’ knowledge, no attempt has been made until now to use a submerged membrane bioreactor with white-rot fungi culture for decolorization of dye wastewater. Water reuse in the textile industry requires appropriate effective treatment process which enables an acceptable water quality (25).

The efficiency of decolorization was studied from the white rot fungi strains through agar plate and liquid batch studies. Subsequently, the feasibility of a submerged microfiltration membrane bioreactor implementing the fungi culture for the treatment of the textile dye wastewater was assessed. However, in order to investigate this fungal potential, most of the scientists have used synthetic effluents in controlled conditions. Of course, the obtained results could give little information on how a fungus could behave in a wastewater treatment plant, competing with bacterial contamination. To date, very few experiments have faced the industrial problematic, so that nowadays the application of fungi in a plant is still a technical challenge.

## 2. Objectives

In the present study, a bench-scale submerged microfiltration membrane bioreactor (MBR) was used to assess the treatment of textile wastewater and its reuse.

## 3. Materials and Methods

The white-rot fungi strains used in the present study, *Coriolus versicolor*, NBRC 9791 and NBRC 30388 were obtained from the NITE biological resource center (NBRC), Japan. The stock culture was grown on potato sucrose agar (PSA) medium at 26.5°C (growth temperature ranging from 24 to 28°C) as prescribed by NBRC. The culture was maintained at 4°C and refreshed every 30-40 days. NBRC 9791 was used in the bioreactor experiment owing to its superior performance in the batch test.

Poly R-478 (polyvinylamine sulfonated backbone with anthrapyridone chromophore, violet color) and Poly S-119 (polyvininylamine backbone with an azo chromophore, orange color) were chosen for the present study. The corresponding absorbance peaks in the visible range for Poly R-478 and Poly S-119 are 520 nm and 472 nm, respectively. Since textile effluent contains a range of dyes, successful decoloration of a single dye does not adequately indicate the suitability of an organism for a decoloration process. However, these two polymeric dyes represent the majority of the synthetic dyes (2).

### 3.1. Degradation and Decoloration Studies

Solid medium in the Petri plates was prepared using PSA medium to which an aliquot of an individual dye was added to a final concentration of 100 mg.L^-1^. Each plate contained one of the aforementioned dyes and a control plate without dye into which NBRC 9791 and NBRC 30388 were inoculated and kept for incubation at 26.5ºC (IL 600 incubator, Yamato). In this study, un-inoculated plates served as controls for abiotic decoloration. The experiment was performed in triplicate for each culture.

### 3.2. Preparation of Liquid Liquid Culture Media

In the present study synthetic medium was used; same as the low nitrogen media optimized by Emrah *et al*. (892007) and G. McMullan *et al*. (2001) for *C*. v*ersicolor*. with a minor modification. The only modification was the replacement of glucose by starch, which is used in the real textile wet processing. The synthetic medium was composed of 4.5 g.L^-1^ starch, 0.4 g.L^-1^ urea, 2 g.L^-1^ KH_2_PO_4_, 0.099 g.L^-1^ CaCl_2_, 1.025 g.L^-1^MgSO_4_.7H_2_O, 0.001 g.L_-1_ thiamine, 1 mL.L^-1^ traceelements, and the desired concentration of the dyestuff. The TOC of the medium was around 2000 mg.L^-1^ (dye TOC ≈ 50 mg.L^-1^). Stock trace elements solution was prepared by dissolving 0.125 g CuSO_4_.5H_2_O,0.05 g H_2_MoO_4_, 0.061 g MnSO_4_.5H_2_O, 0.043 g ZnSO_4_.7H_2_O, and 0.082 g Fe_2_(SO_4_)_3_ .14H_2_O in 1 L of milli-Q water. The pH of the medium was adjusted to 4.5 using HCl and NaOH.

### 3.3. Batch Studies and Degradation Pathway in Aqueous Solution

300 mL flasks containing 200 mL of the culture medium (with 50 mg.L^-1^ of dye) were aseptically inoculated with four cut pieces (approximately 1cm2) from the actively growing culture on an agar plate and incubated at the optimum growth temperature of 28ºC in aerobic condition (air diffusion through silicon stopper) on a shaker (BR-300LF, Taitec Bio-shaker, Japan) at a speed of 150 rpm for the specified period. After inoculation, at the indicated intervals of the incubation, 2 mL of the extracellular culture was removed and diluted properly (5 times for absorbance and 50 times for TOC measurement) with milli-Q water before measurement of the absorbance and Total Organic Carbon.

### 3.4. Equipment and Operating Conditions of the Bioreactor

The Yu-Lan Jin *et al*. (2006), the schematic of the lab-scale submerged MBRs are referred in this study. A laboratory scale membrane bioreactor made of PVC pipes with a working volume of 12.5 liters was used and operating conditions for microfiltration membrane bioreactor (MBRs) are shown in (Table 1). A schematic set-up of the experiment is depicted in (29). To facilitate complete mixing, the reactor was divided by a baffle into two interconnected compartments, the larger one containing two hollow fiber polyethylene membranes (UMF 0234L1, Mitsubishi Rayon), each having a surface area and pore size of 0.2 m2 and 0.4 μm, respectively. The air was provided from the bottom of the reactor by using three diffusers connected to air pumps. The central diffuser (air flow 5 L.min^-1^) and the other two diffusers (air flow from each 2.5 L.min^-1^) were operated alternately with a 5 min cycle so that at any time the‏ aeration rate in the reactor was 5 L.min^-1^. This type of arrangement of the diffusers was expected to be effective for complete mixing along with membrane cleaning. The system was operated continuously under a controlled temperature of 29±1ºC. The pH of the system was controlled at 4.5±2 by adding 0.3 N HCl and 0.3 N NaOH applying the pumps controlled by a pH controller. The media used in the experiment was the same as that was used in the liquid batch test. The pH adjusted concentrated synthetic wastewater was diluted with the pH adjusted tap water and then supplied into the reactor by pumps controlled by a level controller (61F, Omron). The concentrated media was constantly stirred. As well, the temperature of the mixing tank was kept at 50ºC to avoid settling of starch, which is poorly soluble in water at room temperature. The reactor was operated with an average flux of 0.05 m.d^-1^ (hydraulic retention times (HRT)= 15 hrs). This operation rate could produce 20 liters of the effluent every day. The effluent was filtered out through the membranes by suction pumps with a 5 min on/off cycle (an instantaneous flux of 0.1 m.d^-1^ across each membrane) for the first 9 days after which the cycle was changed to 9 min on and 1 min off to reduce the instantaneous flux (0.055 m.d-1) while maintaining the same average flux. The transmembrane pressure was monitored using vacuum pressure gauges (GC 61, JUST).

**Table 1:** Operating condition for MBRs

**Parameters**	**Ranges**
Working volume	12.5 L.day^-1^
Temperature	29±1^O^C
pH	4.5±2
hydraulic retention times (HRT)	15h
Air flow rate	2.51/min
Aeration rate	51/min
mixed liquor suspended solids (MLSS)	2000 mg.L^-1^

The system was first inoculated with fungi grown for two weeks in 1 liter Erlenmeyer flasks each containing 500 mL of the culture media and the reactor was operated in batch mode for a week after which the continuous operation was started with an mixed liquor suspended solids (MLSS) concentration less than 2000 mg.L^-1^. The specific amount of sludge was wasted from the reactor and membranes were cleaned (off site manual cleaning with water) when membrane fouling was so severe that the transmembrane pressure exceeded 60 kpa or so.

### 3.5. Analytical Methods

Total organic carbon (TOC) analyzer (TOC-V, Shimadzu C391-E058K, Germany) was used to analyze TOC as per standard methods (29). The samples for TOC analysis in the batch tests were homogenized (Branson sonifier 450) for 5 min (30% duty cycle, output control of 3) prior to the measurement. The samples were not filtered, as starch which is poorly soluble would be retained by a filtering unit of 0.45 μm. Samples from the effluent of the membrane bioreactor were free from suspended solid and hence did not require any treatment before TOC measurement. The color (Dye) of the samples was measured by using a spectrophotometer (U-2010, Hitachi, USA). The concentration of dyestuff was calculated from a calibration curve of “absorbance versus concentration” and concentration values were used for calculations of decolorization efficiency. The sample from batch test for absorbance measurement was filtered through a Dismic-25 hydrophilic filtering unit (0.45 μm, mixed cellulose ester, Sigma-Aldrich). The absorbance measurement was carried out on the reactor supernatant and the final effluent after centrifuging sample (H-3R centrifuger, Kokusan) for 10 min at 3000 rpm. MLSS concentration was measured according to the APHA standard methods (29).

### 3.6. Experiment of the Treated Effluent for Reuse

The treated wastewater was used in dyeing and finishing experiments on the industrial scale by means of three dyeing machines in the workshop. The dye bath has been prepared according to a dye recipe. Preweighed dye and fresh-water soaked knitted cotton fabric were introduced into the cold dye bath. The dye bath temperature was gradually elevated to boiling and maintained for about 1.5 h, the water level in the dye bath has been maintained during the dyeing processing. The dyed fabric was rinsed three times with recycled water and dried under the shade, then subjected for dye uptake studies. The dye uptake of the fabric was measured using a spectrophotometer.

## 4. Results

**Figure 1:**
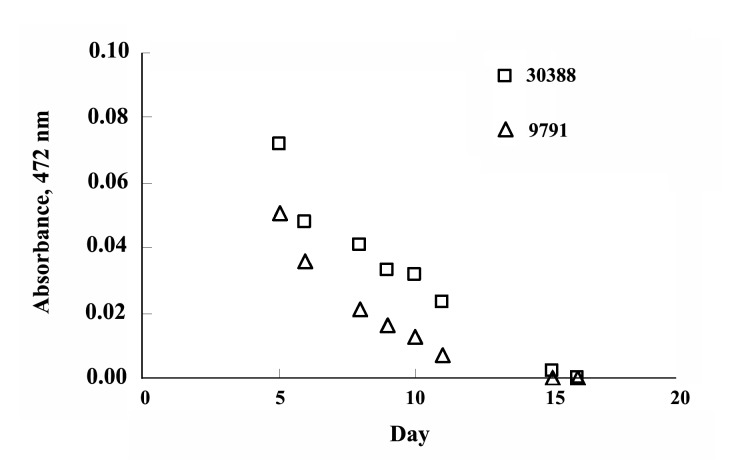
Decolorization of Poly S119 by the two fungi strains (Batch test)

In this study, both the fungi strains showed an appropriate growth in the liquid medium and a stable decrease of the absorbance value was observed and almost complete disappearing of the absorbance has happened within two weeks (Figure 1).‏

**Figure 2:**
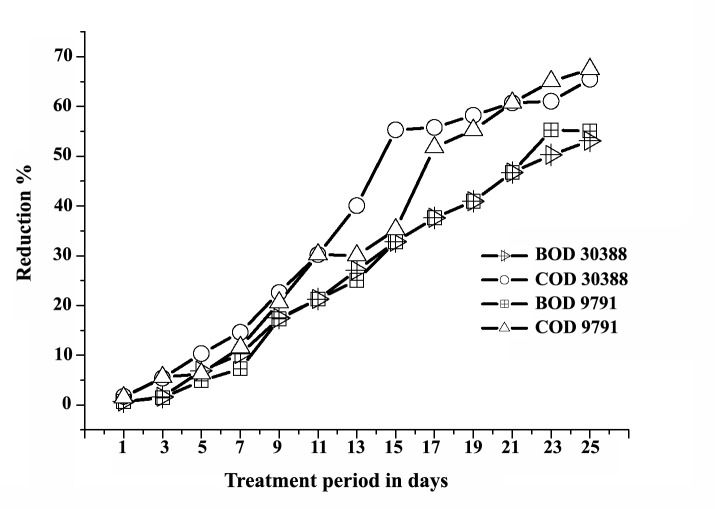
BOD and COD removal by the fungi strains -30388 and 9791 (Batch test)

**Figure 3:**
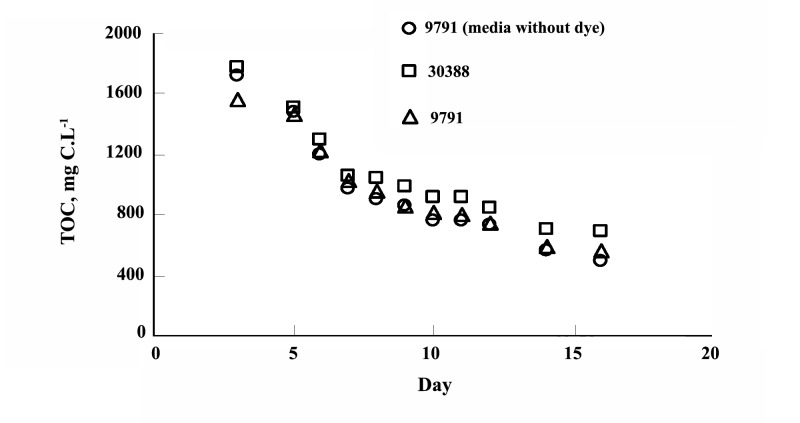
TOC removal by the two fungi strains (Batch test)

**Figure 4:**
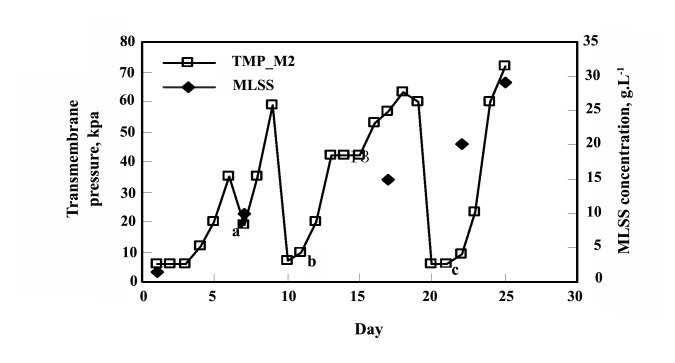
Variation in the MLSS concentration versus transmembrane pressure through the membrane. a: Membrane cleaning, b: Sludge withdrawal and suction cycle change, c: Membrane cleaning and sludge withdrawal

The experiments shown that maximum 53.06% of biological oxygen demand (BOD) and 67.47% of chemical oxygen demand (COD) can be removed by stain-30388 after 25 days (Figure 2) and whereas stain- 9791 can reduce upto 55.06% and 67.47% subsequently (Figure 2). The result shown one-fourth of initial TOC of approximately 2000 mg C.L^-1^ was reduced 2 weeks (Figure 3). The removal efficiency of TOC by the reactor ranged from 92% at the beginning to 97% after a week, and further on. After the operation period the effluent TOC never exceeded 160 mg.L^-1^; the average of which (i.e. effluent TOC) was around 70 mg.L^-1^. The result also shown the supernatant TOC was around 500 mg.L^-1^ (includes fungal TOC). The initial MLSS concentration of the reactor was less than 2 g.L^-1^, it was gradually increased and despite of total sludge wastage of 7 liters in two steps (on 10th and 20th day) and the MLSS concentration rose up to 29 g.L^-1^ (Figure 4) within 25 days. The concentration of dyestuff as calculated from a calibration curve of ‘absorbance versus concentration revealed nearly complete (25 days) decolorization (98%).

**Figure 5:**
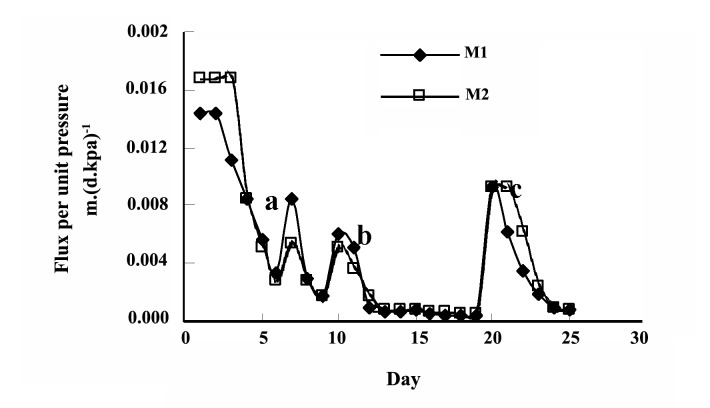
Variation of flux per unit pressure through the membranes a. Membrane cleaning, b. Sludge withdrawal and suction cycle change, c. Membrane cleaning and sludge withdrawal. M1 and M2: Variation of flux per unit pressure through the membranes

The dye uptake of the fabric was measured using a spectrophotometer and has been reported that K/S values using reclaimed water lay within the limit compared with the results obtained using tap water. The study was also found that 3 out of 25 samples were of less than the acceptable level according to color difference (ΔE) value of the dyed samples from the treated wastewater. Unacceptable results were found only when dyeing by very light colors was tested.

## 5. Discussion

The preliminary assessment of the dye decoloration was completed using a solid medium. After five days, the study has shown that the extent of mycelial growth on the agar plates was similar for all cultures whether or not any dye was present and fungi grew extensively with white mycelia all over the agar plates. Both the top and bottom of the agar plate appeared almost colorless when the over-grown fungi mycelia on top of it were carefully removed after 20 days. It was the clear signal of decoloration capacity of the two strains studied here. No abiotic decoloration was observed in uninoculated plates.

In this study, both the fungi strains showed an appropriate growth in the liquid medium, although the growth of NBRC 9791 was a bit faster. The fungi grew like white cotton balls in colorless culture media, and in media with dye, the fungi mycelia turned colored due to absorption of the dye. Figure 1 shows the decoloration of Poly S-119 by both the fungi strains. A stable decrease of the absorbance value was observed and almost complete disappearing of the absorbance has happened within two weeks. The study also showed that decoloration rate of NBRC 9791 is faster than that of NBRC 30388 and the absorbance value did not‏ increase further even in 1 month which indicated stable decolorization without any release of dye from the fungi mycelia. Similarly, efficient decoloration was exhibited in t he case of the dye Poly R-478 (results not shown).

The fungi have also shown stable TOC removal. The initial TOC of approximately 2000 mg C.L^-1^ was reduced to almost its one-fourth within 2 weeks (Figure 3). The TOC removal by the fungi in the medium‏ with or without dye was analogous, which indicated‏ the suitability of the fungi for colored wastewater‏ treatment. Over a month, the final pH of the media‏ inoculated with NBRC 30388 and NBRC 9791 were‏ 5.61 and 5.3, respectively‏.

### 5.1. Performance of the Membrane Separated Fungi Reactor

The initial MLSS concentration of the reactor was‏ less than 2 g.L^-1^, it was gradually increased and despite‏ of total sludge wastage of 7 liters in two steps (on 10^th^‏ and 20th day) and the MLSS concentration rose up to‏ 29 g.L^-1^ (Figure 4) within 25 days. Transmembrane‏ pressure was increased sharply (Figure 5) due to‏ severe membrane fouling by the fungi. The filamentous‏ fungi were entwined with the membrane fibers in‏ such a way that the fine air bubbles from the diffusers‏ could not scrub the fungi off the membrane; rather the‏ bubbles sometimes pushed the fungi more into the‏ membrane (30). In this case, a flat sheet type membrane‏ module, with its characteristic flat shape and‏ fiber less structure, might be suitable to prevent excess‏ membrane attachment of the fungi. There is also an‏ opportunity to improve the design of the reactor and‏ the aeration system to ensure enhanced mass transfer‏ and scouring of sludge from the membrane surface‏ (31, 32-35). The membranes were cleaned twice, first‏ on day 7 (inside the reactor simply by brushing) and‏ then on day 20 (outside of the reactor simply by water)‏ during the 25 days operation period. Higher ‘flux per‏ unit pressure’ (Figure 5) was observed at the initial‏ stage and decreased later on. The ‘flux per unit pressure’‏ was recovered to some extent by membrane‏ cleaning (day 7), suction cycle change and sludge‏ withdrawal (day 10), and simultaneous cleaning and‏ sludge withdrawal (day 20). After around three weeks,‏ the fungi culture was observed to be predominately‏ composed of fine particulate pellets rather than filamentous‏ ones. However, this change did not influence‏ the transmembrane pressure or color and TOC‏ removal.

### 5.2. Removal of Total Organic Load

The reactor efficiency of treating wastewater at various‏ organic loading rates was studied and it was performance‏ well. The reduction efficiency of BOD and‏ COD was upto 50 to 70% of their initial load (Figure 2). The removal efficiency of the total organic carbon‏ (TOC) by the reactor ranged from 92% at the beginning‏ to 97% after a week, and further on. Figure 3 shows the‏ TOC variation in the effluent during the operation period.‏ The TOC of the influent water was around 2000‏ mg.L^-1^, after the operation period the effluent TOC never‏ exceeded 160 mg.L^-1^, the average of which (*i.e.* effluent‏ TOC) was around 70 mg.L^-1^. The supernatant TOC was‏ around 500 mg.L^-1^ (includes fungal TOC). The major‏ portion of the influent TOC was contributed by the high‏ dose of starch, and the membrane used in this study (pore‏ size 0.4 μm) was able to retain a considerable portion of‏ the poorly soluble starch by sieving. In fact, starch was‏ observed to be adsorbed on the surface of the membrane‏ attached fungi and create a sticky layer on the membrane.‏ Also, an amount of starch was observed to settle‏ at the bottom of the reactor. However, there was no gradual‏ accumulation of the settled starch, which indicates its‏ subsequent degradation and assimilation by the fungi.

### 5.3. Degradation and Decolorization of Dye

**Figure 6:**
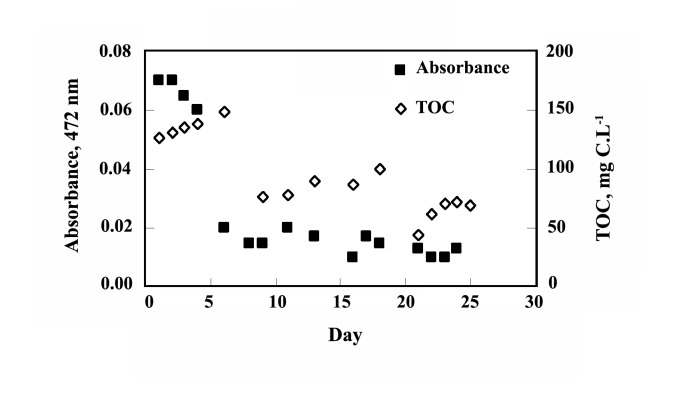
TOC and color removal by the reactor (Influent TOC≈200 mg C/L^-1^, absorbance=3)

**Figure 7:**
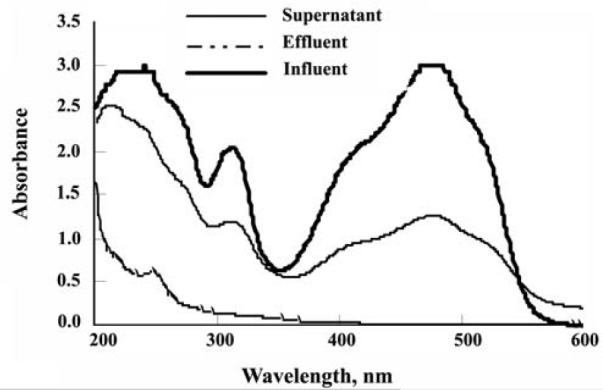
A comparative analogy of the UV-visible spectra showing the simultaneous decolorization and biodegradation of azo dyes

The reactor showed stable decolorization throughout‏ its operation period. The concentration of dyestuff‏ as calculated from a calibration curve of ‘absorbance‏ versus concentration’ revealed nearly complete (25‏ days) decolorization (98%) (Figure 6). Degradation‏ and decolorization of Poly S-119 were also followed‏ by analysis of UV-VIS absorbance scanning before‏ and after the treatment. The UV-VIS spectrum of the effluent of the bioreactor showed a remarkable change‏ after the treatment (Figure 7). The disappearance of the‏ absorbance peak at 472 nm indicated an unequivocal‏ signal of the nearly complete decolorization and the‏ breakdown in the chromophoric group. Besides, the‏ notable diminution of the absorbance peak at 210 nm‏ is related to the cleavage of the aromatic group present‏ in the original structure of the dye (10).‏

In this study, the dye Poly S-119 itself was soluble‏ enough to pass through the microfiltration membrane‏ (pore size 0.4 μm) used. This, however, was observed‏ to be retained by a laboratory Dismic-25 filtering unit‏ (0.45mm) when mixed with starch to make the synthetic‏ wastewater. From this notion, it might be stated‏ that the membrane first retained the major portion of‏ the dye adsorbed on starch and on the fungi, then, the‏ fungi subsequently degraded the dye (34, 35). Asynergistic‏ effect of the membrane-fungi-starch combination‏ for decolorization is thus projected here.‏

### 5.4. Alternative Water Source for Dyeing and Finishing Processes

The treated wastewater can be used in the workshop.‏ The dyed fabric was rinsed three times with‏ recycled water and dried under the shade, then subjected‏ for dye uptake studies and observed that K/S values‏ using reclaimed water lay within the limit compared‏ with the results obtained using tap water. The study‏ found from 50 random samples that there was no difference‏ in the washing fastness between dyed cotton‏ cloths that were washed with the reclaimed water as‏ compared with fresh water. This study suggested that‏ the wastewater could be divided into two parts, one‏ (COD concentration of over 500 mg.L^-1^) will be discharged‏ into the wastewater treatment plant; the other‏ (COD concentration of less than 500 mg.L^-1^) can be‏ discharged into the experimental tank (35). The results‏ from the experimental work showed that the reclaimed‏ water may be used for production excluding the last‏ rinse. The dyed fabric was last rinsed with tap water.

## 6. Conclusions

In this study, the preliminary agar plate and aqueous‏ batch decoloration investigations have revealed‏ the decoloration efficiency of the fungi strains (*C. versicolor*)*.* The stable TOC and color removal (>95%‏ and 98%, respectively) from the synthetic wastewater‏ by the submerged microfiltration membrane reactor‏ using the white rot fungi (*C. versicolor*, NBRC 9791)‏ presents the system as a promising one. The synergistic‏ effect of the starch-fungi-membrane combination‏ on decolorization would be of the special interest.‏ Membrane fouling rate is required to be subject of further‏ study before recommending the MBR with spiral‏ wound model as a technique of choice for water reuse.‏ In conclusion, a very interesting fungal strain, was‏ selected for its capability to be active in bioremediation‏ processes, acting towards several parameters, as‏ colour, TOC, BOD and COD. A complementary‏ approach with active sludge could be hypothesized.‏ From a practical point of view, in the future, it should‏ be considered to evaluate the fungal potential also during‏ longer treatment, carried out on several cycles, in‏ order to mimic the industrial conditions the fungus‏ would work in. Furthermore, the process should be‏ scaled-up to larger volume, in order to confirm the‏ robustness and the applicability of the system.‏

## Acknowledgements

The authors acknowledge The World Academy of‏ Science (TWAS) Italy, and Universiti University Saints Malaysia, Malaysia, for providing world class‏ infrastructure for continuing the research work.
